# Symbiotic cooperation between freshwater rock-boring bivalves and microorganisms promotes silicate bioerosion

**DOI:** 10.1038/s41598-020-70265-x

**Published:** 2020-08-07

**Authors:** Damien Daval, François Guyot, Ivan N. Bolotov, Ilya V. Vikhrev, Alexander V. Kondakov, Artem A. Lyubas, Andrey Y. Bychkov, Vasily O. Yapaskurt, Martiane Cabié, Oleg S. Pokrovsky

**Affiliations:** 1 Laboratoire d’Hydrologie et de Géochimie de Strasbourg, UMR 7517, Université de Strasbourg–CNRS/ENGEES–EOST, Strasbourg, France; 2 Institut de Minéralogie, de Physique des Matériaux et de Cosmochimie, Museum National d’Histoire Naturelle, Sorbonne-Université, UMR 7590 CNRS, Paris, France; 3 Institut Universitaire de France, Paris, France; 4 N. Laverov Federal Center for Integrated Arctic Research, The Ural Branch of the Russian Academy of Sciences, Northern Dvina Emb. 23, 163000 Arkhangelsk, Russia; 5 Lomonosov Moscow State University, Faculty of Geology, Leninskiye Gory, 1, 119991 Moscow, Russia; 6 Aix-Marseille Université, CNRS, Centrale Marseille, FSCM, CP2M, Marseille, France; 7 Geosciences and Environment Toulouse, UMR 5563 CNRS, Toulouse, France; 8 BIO-GEO-CLIM Laboratory, Tomsk State University, Tomsk, Russia

**Keywords:** Biogeochemistry, Geochemistry, Geology, Geomorphology, Biogeochemistry

## Abstract

Bioerosion is a process with a high socio-economic impact that contributes to coastal retreat, and likely to increase with climate change. Whereas limestone bioerosion is well explained by a combination of mechanical and chemical pathways, the bioerosion mechanisms of silicates, which are harder and chemically more resistant, remain elusive. Here we investigated the interface between siltstone and freshwater rock-boring bivalves *Lignopholas fluminalis* (Bivalvia: Pholadidae). Remains of a microbial biofilm were observed only in the poorly consolidated part of the rock within the macroborings created by bivalves. Secondary Mn-bearing minerals identified in the biofilm suggest that microbes promoted silicate rock weathering by dissolving Mn-rich chlorites. Moreover, hard mineral debris found in a biofilm attached to the shells likely contributed to the abrasion of the rock substrate. Thus, beyond the classical view of chemical and/or mechanical action(s) of macroborers, silicate bioerosion may also be facilitated by an unexpected synergistic association between macro- and microorganisms.

## Introduction

Bioerosion is a commonplace strategy developed by living organisms, which consists in boring hard substrates of various origins, including biological materials (e.g., wood, shells, and bones)^1^, mud^2^, rocks^3^ and even synthetic materials. Depending on the nature of the substrate and the borer, bioerosion ensures a wide range of metabolic activities and ecosystem services, ranging from nutrition^4^ to the creation of microhabitats protected from predators for themselves as well as for secondary dwellers^5^.

Gaining knowledge into the occurrence, rates and mechanisms of boring is of fundamental importance for a series of reasons. First and historically, mankind has been confronted with macroborers through the damages caused by shipworms on vessels, wooden wharfs and docks^5,6^. More broadly, bioerosion has socio-economic impacts whenever manufactured materials are damaged, including plastic, metals and concrete materials such as levees or coastal defences^3,6^. Second, bioerosion contributes to element recycling, shaping landscapes through the weakening of rocky shorelines and participating to coastal retreat^5^, for which the current rates are likely to be modified drastically as a result of climate change^6^. Third, the creation of microhabitats by macroborers such as bivalves is correlated with a significant increase of the abundance of species assemblages, thus partly contributing to local faunal biodiversity^7^. Finally, fossil records of macro-bioerosion may be used as a biological proxy to estimate the paleo-location of intertidal and shallow subtidal marine environments, marking ancient shorelines^8^.

The mechanisms of rock bioerosion associated to macroborers and especially bivalves have been a source of lively debate for decades, and can be schematically divided into two main pathways. First, rock boring can occur chemically through biocorrosion (also referred to as bioweathering). It is generally admitted that chemical etching is only possible when boring occurs in (at least partly) calcareous substrates^5^, as most of the common biologically secreted agents by macroborers exhibit a modest impact on the dissolution rate of silicate minerals^9^. The biologically produced substances that promote the dissolution of carbonates include a variety of lipoproteic components with acidic groups, CO_2_, or calcium-binding mucoproteins secreted by pallial glands, located along the edge of the mantle of chemically boring bivalves^5,10,11^. Biocorrosion was then suggested to proceed along grain boundaries first, prior to affect bulk minerals^5^. Second, rock boring can also occur mechanically through bioabrasion. Several lines of evidence support this mechanism, including (i) the microtexture of the shells, consisting of excavating ridges and rasping structures rendering bivalves comparable to a so-called “living lime”^12^, (ii) muscular adaptation, as described by Shipway et al.^4^ regarding the large posterior adductor muscle of the shipworm *Lithoredo abatanica* (Bivalvia: Teredinidae), (iii) experimental and modeling results of the stimulation of the muscles that control the rotation of the shells of the bivalves, which made it possible to accurately reproduce the shapes of burrows and scrape marks resulting from the boring by the bivalve *Barnea candida* (Bivalvia: Pholadidae)^10^. Finally, it is now generally admitted that both mechanical abrasion and chemical etching occur synergistically, especially for living organisms that bore in calcareous rocks, where the substrate is first weakened chemically, while individual grains are subsequently excavated mechanically from the boring^5,9^.

The efficacy of both biocorrosion and bioabrasion is all the more intriguing when it comes to consider boring in silicate rocks such as siltstone^8,9^, gneiss^13^, basalts^3^ or quartzite^14^, whose hardness is sizably higher than that of the aragonite/calcite shells of the borers. The mechanisms proposed to account for these observations include (i) the possible softening of the rocks, prior to the onset of macroboring, by microendolithic organisms^14^, capable to create microchannels in silicate materials such as basaltic glass^15^, (ii) the exploitation of differences in mineral hardness and crystal boundaries in the rock (see^3^ and references therein), (iii) a modification of the shell morphology, which may be appropriate to bore into very hard substrates^12^. However, these mechanisms essentially remain hypothetical, for which direct evidence are most often lacking.

**Figure 1:**
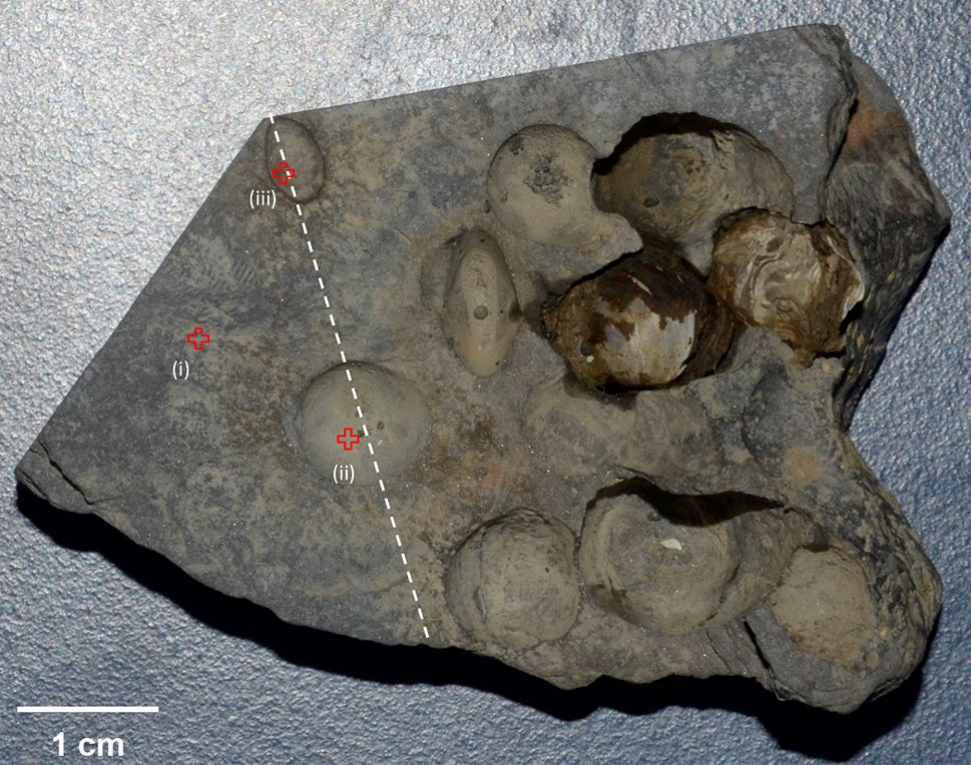
Photograph of a piece of siltstone collected from the middle reaches of the Kaladan River, western Myanmar, showing extensive pitting by *Lignopholas fluminalis* bivalves. The sample has been cut following the dashed line, and three locations were investigated by electron microscopy, namely (i) an area devoid of macroborings, the bottom of (ii) a large and (iii) a small macroboring, respectively.

The present study was designed to provide new insights into these questions. Recently, ongoing boring in siltstone rocks from a freshwater section of the Kaladan River in Myanmar (Fig. 1) by *Lignopholas fluminalis* (Bivalvia: Pholadidae) bivalve has been identified^8^. The boring mechanisms were not revealed in this previous study, but were suggested to be comparable to those used by their marine relatives in softer substrates^10^, i.e., bioabrasion. Here, we combined analytical scanning electron microscopy (SEM) and focused ion beam (FIB) milling coupled to transmission electron microscopy (TEM) to document the chemical and mineralogical compositions of the host rock and of the shell-rock interface at the micro- to nanometer scale. In addition to the possible bioabrasion mechanisms proposed by Bolotov et al.^8^, our results suggest that the boring may be microbially-assisted in a symbiotic-like process, where microbes would take advantage of loosening the mineral grains to dissolve minerals cementing the siltstone and containing essential elements for their growth, ultimately resulting in the weakening of the substrate, facilitating further bioerosion by the bivalves.

## Results

### Distinctive features of the surface of the rock substrate devoid of macroborings

**Figure 2:**
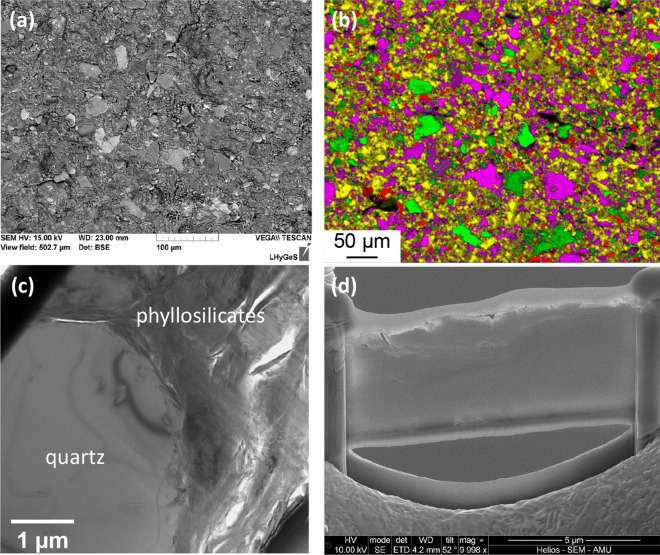
Electron microscope characterizations of the rock sample at location (i), where the surface is devoid of macroborings. (**a**) BSE image of the surface obtained using SEM, showing the typical assemblage of silicates debris making up the siltstone. (**b**) Color map obtained from EDX analyses, showing the random distribution of minerals making up the siltstone at the sub-mm scale. Green, red, purple and yellow colors represent minerals enriched in Mg (chlorite), Na (feldspars), K (feldspars, clays) and Si (quartz, clays), respectively. Black pixels essentially refer to voids (porosity) in the rock. (**c**) TEM image of the FIB thin section shown in (**d**). The grain assemblage appears much more compact than that observed at the bottom of the macroborings (see Fig. 4a,b).

At the submillimeter scale, the rock-forming minerals appear homogeneously distributed, devoid of any layering or of specific mineral patches (Fig. 2a,b). The most abundant minerals, i.e., quartz, feldspars and clays are randomly distributed, and heterogeneous clusters could not be evidenced. Statistical analyses of color maps such as shown in Fig. 2b indicate that the surface is covered with ~ 63% of quartz + clay minerals; ~ 25% feldspars (note that the proportion of feldspars is most likely overestimated due to possible mistaken assignment of the Kα and Kβ emission lines in the EDX spectra to K-feldspar instead of illite); ~ 10% chlorite; 3% voids (porosity). These results are in reasonable agreement with the analyses reported by Bolotov et al.^8^ (see “Materials and methods”). The homogeneous distribution of minerals was also revealed at the submicron scale on the FIB foils that were extracted from the “pristine” (i.e., devoid of macroborings) area (Fig. 2c). Imaging the rock substrate in cross-section further indicates that the grains display a packed and cohesive arrangement, as further suggested by the fact that individual grains did not tend to pop out during the FIB milling procedure (Fig. 2d).

### Distinctive features of the rock surface located in macroborings

**Figure 3:**
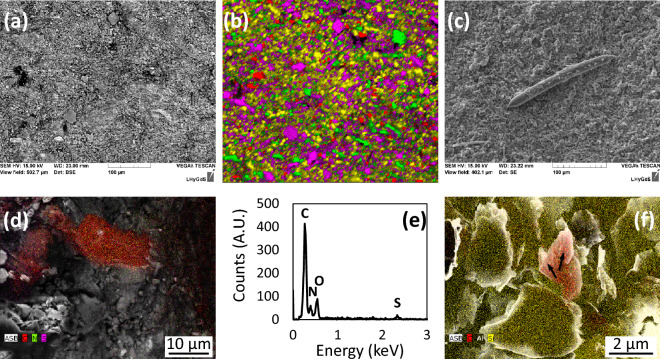
Electron microscope characterizations of the rock sample referring to the bottom of the largest macroboring (location (ii) in Fig. 1). (**a**) BSE image of the surface obtained using SEM, showing the typical assemblage of silicates debris making up the siltstone. (**b**) Color map obtained from EDX analyses, showing the random distribution of minerals making up the siltstone at the sub-mm scale. Green, red, purple and yellow colors represent minerals enriched in Mg (chlorite), Na (feldspars), K (feldspars, clays) and Si (quartz, clays), respectively. Black pixels refer to voids (porosity) in the rock. (**c**) SEM image of a needle made of pure amorphous silica and interpreted as a megasclere of *Corvospongilla ultima* sponge. (**d**) SEM image of the bottom of the macroboring with superimposed EDX chemical analyses (C, N and S are represented by red, green and purple colors, respectively). (**e**) EDX spectrum of the C-rich location shown in (**d**). (**f**) SEM image of the remains of putative bacterial cells (arrows) with superimposed chemical analyses (C, Al and Si are represented by red, black and yellow colors, respectively).

At the submillimeter scale, the distribution of the rock-forming minerals in the macroborings appears homogeneous and cannot be distinguished from the distribution of minerals in locations devoid of macroborings (Fig. 3a,b). Statistical analyses of color maps such as shown in Fig. 3b indicate that the surface is covered with ~ 51% of quartz + clay minerals; ~ 28% feldspars (most likely overestimated, see above); ~ 13% chlorite; 6% voids (porosity). No specific abrasive imprints could be evidenced in the macroborings, as opposed to other studies that have reported the identification of scrap marks or concentric grooves left in the boreholes, resulting from the rotation of the valves of mechanical borers^3,10^. It is unlikely that such features would have faded away because of later erosion here, since these observations also apply to macroborings still filled with mollusks.

Patches of several µm^2^ of a (C, N, S)-rich organic matrix interpreted as the remains of a complex microbial biofilm were found only in the macroborings (Fig. 3d,e), which also exhibited several bacteriomorph structures approaching the size, shape and chemical composition of bacterial cells (Fig. 3f). Finally, 100-µm long needles were occasionally observed in some of the macroborings (Fig. 3c). These needles are made of pure amorphous silica and resemble in size and shape the megascleres of sponges such as *Corvospongilla ultima* (Demospongiae: Spongillidae) described in^16^. This observation is consistent with the results of Bolotov et al.^8^, who reported that *Corvospongilla ultima* was among the nestling species associated with *Lignopholas fluminalis*’ ecosystem.

**Figure 4:**
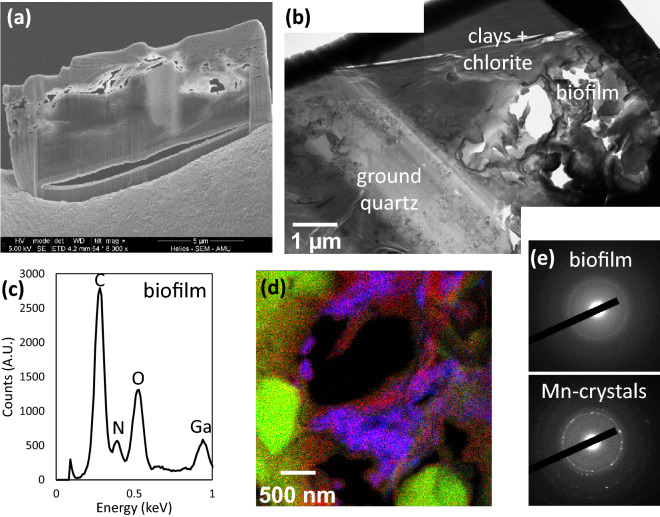
TEM characterizations of a FIB thin section excavated from the bottom of a small macroboring (location (iii) in Fig. 1). (**a**) SEM image of the FIB thin section, showing the poorly consolidated assemblage of minerals in the area that was contacting the bivalve. (**b**) TEM image of the FIB thin section, showing ground quartz and an area enriched in C and N (labeled “biofilm” on the figure). (**c**) EDX spectrum of the biofilm. (**d**) EDX color map of the biofilm area shown in (**b**). Mn, Si and N are represented by blue, green and red colors, respectively. Note the occurrence of nanosized quartz debris. (**e**) Selected area electron diffraction of the amorphous biofilm and the Mn-bearing crystals.

At the submicron-scale, the chemistry and texture of the substrate at the bottom of the two macroborings significantly differ from that described in locations devoid of macroborings. The assemblages were made of low consolidated grains which tended to pop out during the FIB milling procedure (Fig. 4a). Ground silicate grains were observed (Fig. 4b), and µm-size voids filled with an amorphous (C, N)-rich matrix were evidenced in each of the two FIB thin sections (Fig. 4c–e). This matrix was enriched with Mn- and Ca-bearing nanocrystals (Fig. 4d,e). However, the values of the interplanar spacing estimated from the electron diffraction patterns were too short (comprised between 0.91 and 2.24 Å) to be unambiguously attributed to a given specific mineral or mixture of minerals, such that their exact nature remains unknown.

### Chemical and mineralogical characterizations of the interface between shells and siltstone

**Figure 5:**
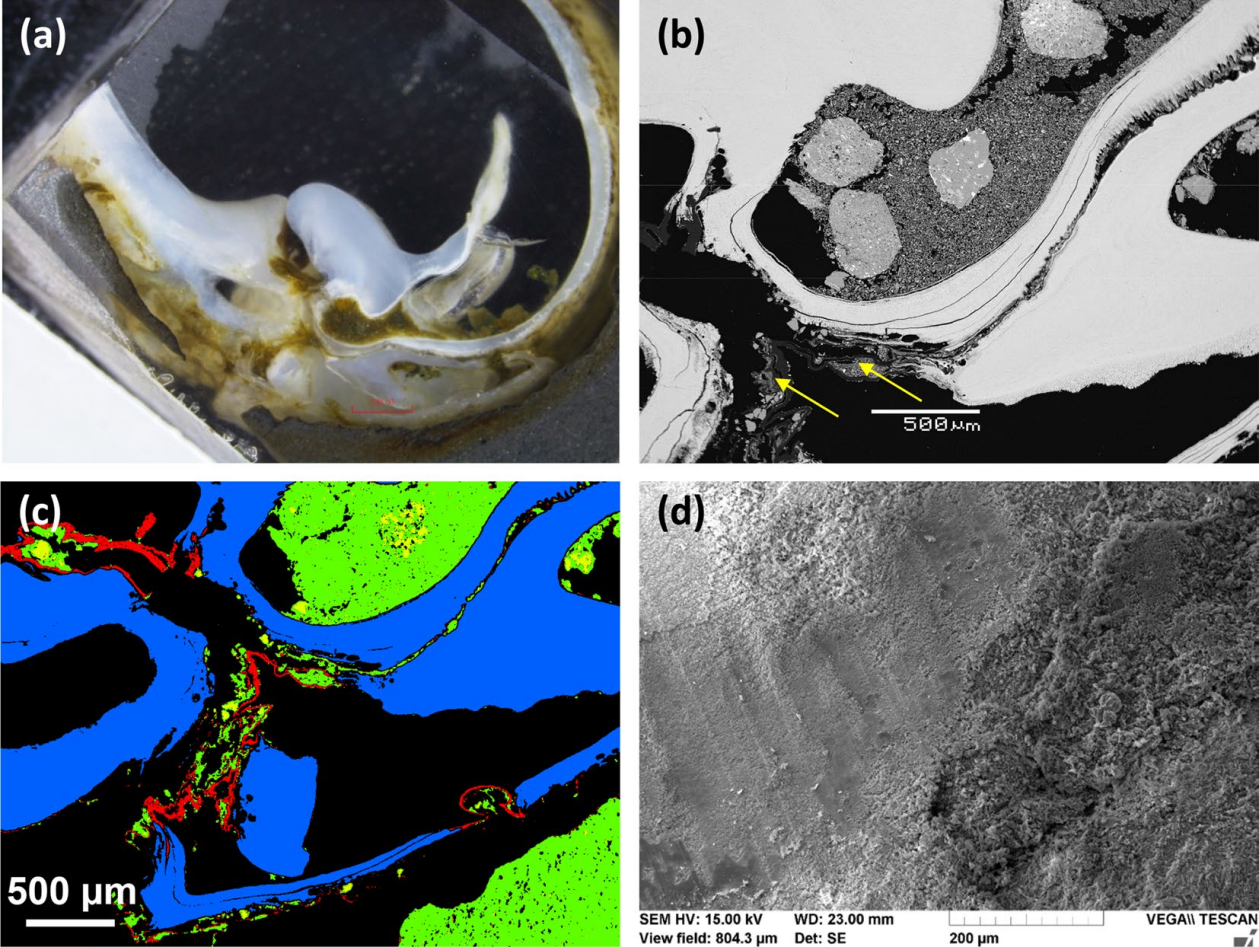
Analyses of the cross section of the bottom part of the shells of a bivalve. (**a**) Photograph of the cross-section imaged in visible light. (**b**) BSE image of the cross-section of the shells. Aragonite is white, quartz and clays are grey, epoxy is black. The arrow indicates the organic film (see also Fig. S3), which contains debris or hard minerals such as quartz and feldspars. (**c**) Integral map of organic and mineral distribution at the shell-siltstone interface. The aragonite that makes up the shell is blue, silicate materials are green and the organic film is red. (**d**) SEM image of the shell of a bivalve. No specific morphological microtexture that could have provided the bivalve with an efficient excavation ability can be seen.

SEM analyses of the transversal cross-section of the aragonite shell–siltstone interface revealed that the shells of *Lignopholas fluminalis* were extensively covered with debris of hard minerals from the siltstone, mainly quartz, feldspars and occasionally, accessory minerals such as rutile (Fig. 5a–c, and SI Appendix Figs. S1, S2 and Table S1). Because the hardness of these minerals ranges from 6 to 7, they can easily bore into the phyllosilicates of the siltstone (chlorite and sericite) which have a hardness of 2 to 3.

Interestingly, these minerals were not found to be simply deposited on the surface of the shells, but literally fixed to the surface via an organic film enriched in nitrogen (20 wt%) and sulfur (4 wt%) (SI Appendix, Fig. S3 and Table S2), which may be interpreted as extracellular polymeric substances (EPS) resulting from microbial activity. The association between carbonate shell, siltstone, and organic matrix with embedded silicate and quartz grains is visualized on integral map of component distribution shown in Fig. 5c. The nitrogen-to-sulfur ratio may reflect the presence of 10% S-containing amino acids such as cysteine in these EPS/proteins.

### Morphological and chemical characterizations of the shells

At the µm-scale, it was not possible to identify a specific morphological microtexture of the shell that would have provided the bivalve with an efficient excavation ability (Fig. 5d). Moreover, the extensive coverage of the shells with exogenous minerals considerably complicated the direct observation of fine scale morphology, so that features such as reported by e.g. Fang and Shen^12^ or Nederlof and Muller^10^ might have remained obscured.

The chemical composition of aragonite that makes up the shells is very homogenous, whilst exhibiting an enrichment in Sr at the extreme point of the shells (SI Appendix, Fig. S4). Total chemical analyses further revealed that the shells of *Lignopholas fluminalis* were slightly depleted in a number of elements compare to the non-boring species from the same location (SI Appendix, Table S3). The average ratio of element concentration in borers (*Lignopholas fluminalis*) to non-borers (*Scaphula deltae*; Bivalvia: Arcidae) demonstrate slight (ca. a factor of 1.2 to 1.5) depletion of borers in Li, Mg, Al, Si, P, K, V, Cr, Mn, Fe, Ni, Zn, Ga, Ge, As, Se, Rb, Cs, Tl, Th. This depletion was not significant because the variations of chemical composition among individual shells were as high as 30%. The average concentrations of Ti, S, Ca, Sr, Cu, Mo and Pb were practically identical (± 5%) in both species, whereas Y and REEs were within 10% similar. The concentrations of Cd, Nb, Hf, W and U were a factor of 2.7 to 6 higher in non-borers compared to borers, whereas B and Ba were a factor of 4.0 and 1.6 higher in borers compared to non-borers. Given the similarities between the chemical composition of the shells of boring and non-boring species, it can be assumed that the shells of *Lignopholas fluminalis* do not possess any chemical specificity that might have made them more prone to boring into hard substrata.

## Discussion

Overall, the characterizations detailed above point out that the contact between the bottom of the macroborings and the shells of the bivalves likely have represented a hotspot of microbial activity, which was not observed elsewhere at the surface of the siltstone devoid of macroborings. In the next two sections, we discuss how the association between *Lignopholas fluminalis* and microorganisms may have acted symbiotically to facilitate boring in siltstone.

### A possible strengthening of the mechanical abrasion thanks to microbial EPS

As mentioned above, macroborings resulting from bioerosion are most often observed either in calcareous rocks, which are highly sensitive to bioweathering, or in soft substrates such as peat or clays, which are readily drilled through bioabrasion. Here, the siltstone is both chemically much more resistant than carbonates and harder than the substrates commonly subjected to bioabrasion.

Bolotov et al.^8^ have reported that the mean hardness of the siltstone was 62 kgf mm^−2^, i.e., twice as much as clayey materials. In comparison, the compilation of Yang et al.^17^ indicates that the hardness of Bivalvia shell is an order of magnitude lower than that of quartz, and only slightly greater than that of the siltstone, ranging between 110 and 270 kgf mm^−2^. In addition, both the structure and hardness of the siltstone were found to be homogeneous, such that it is unlikely that *Lignopholas fluminalis* took advantage of any local weakness to bore into the rock. Finally, the macroboring walls did not exhibit any marks, as opposed to the experimental results obtained by Nederlof and Muller^10^ using the piddock *Barnea candida*, which is a close relative of *Lignopholas fluminalis*^8^. However, such scrap marks resulting from the abrasion of the substrate by the denticles of the piddocks were obtained by rotating the shells in a soft materials (wax). The bioabrasion ability of the shells of *Barnea candida* is thought to be limited to soft substrata such as clays or peat and most likely, they cannot abrade harder substrata such as chalk^10^.

Similarly, we argue that the various features collected here suggest that there is no clear evidence that the direct contact between the shells of *Lignopholas fluminalis* and the substrate is responsible for the bioabrasion of the siltstone. Instead, single grains excavated from the borehole partly remained trapped at the surface of the shells, embedded into an organic matrix that we interpreted as a biofilm. It can reasonably be assumed that these single grains, which are essentially hard minerals such as quartz and feldspars, acted like abrasive materials that contributed to drill the siltstone through the rotation of the shells. Therefore, the presence of microorganisms in the interfacial region between the substrate and the borers possibly strengthened their boring ability, although at that point, it remains impossible to state whether this interaction is obligatory or facultative. In any case, from a mechanical standpoint, *Lignopholas fluminalis* bivalves likely took advantage of the biofilm attached to the surface of their shells to increase their boring ability.

### Enhancing the weakening of the rocks through microbially-induced weathering

In addition to bioabrasion, some macroborers are also known for their ability to promote bioweathering^5^. This mechanism of bioerosion is suggested to be limited to calcareous substrates and not significant for substrates such as siltstones, whose rock-forming minerals have a dissolution rate that is between 6 and 8 orders of magnitude lower than that of calcite at circum-neutral pH conditions (according to rate data from^18^ for quartz^19^, for albite^20^, for chlorite and^21^ for calcite).

Notwithstanding, we argue that mass transfer did occur during the process of boring discussed in the present study. We detail below the reasons why we think that this mass transfer cannot result from the abiotic dissolution of the grains by the bulk fluid, and suggest that microorganisms were responsible for the dissolution of the siltstone, which ultimately facilitated the formation of borings by *Lignopholas fluminalis*.

The strongest evidence for mass transfer is the occurrence of secondary Mn-rich crystals found embedded in an organic matrix at the bottom of the macroborings. Because such minerals were not found elsewhere in the rock sample, this finding indicates that the contact region between the bivalves and the siltstone was not simply mechanically eroded, but also chemically weathered. The source of Mn is most likely chlorite, which represents the richest source of Mn among the rock-forming minerals (0.2 to 0.7 wt% according to quantitative EDX analyses). In addition, the location of the minerals (specifically embedded in the organic matrix) indirectly suggests that microbes were responsible for the dissolution of chlorite. This latter assertion can be further supported by comparing the residence time of a chlorite grain at the bottom of a pit to the time required to dissolve chlorite with a bulk aqueous fluid:

First, several studies estimated the lifespan of bivalve piddocks of the family Pholadidae (to which *Lignopholas fluminalis* belongs) to be on the order of 10 years^22^. The deepest macroborings that we observed, possibly corresponding to the oldest bivalves, were on the order of 1 cm, leading to a mean erosion rate of *R*_*erosion*_ = 1 mm yr^−1^.

Second, the grain size of the siltstone is comprised between 0.2 and 50 µm, with an average value around Ø = 10 µm^8^. The average time (*t*) required for a 10-µm grain to be excavated from the bottom of the pit and released to the environment can thus be estimated following:

1 $$t= \O \cdot {{R}_{erosion}}^{-1}$$

yielding *t* = 10^–2^ year. This value indicates that Mn must be efficiently released from chlorite over a time interval as short as 10^–2^ year (~ 3.7 days) to be incorporated into secondary minerals.

Finally, the radial retreat (∆*h*) of a hypothetical spherical grain of chlorite dissolved over a time interval of 3.7 days, can be calculated using:

2 $$\Delta h= \frac{M}{\rho }{R}_{chlorite} \cdot t$$

where *M* , *ρ*, and *R*_*chlorite*_ stand for the molar mass, the density and the dissolution rate of chlorite, respectively. Considering the rate data from Lowson et al.^20^, the far-from-equilibrium dissolution rate of chlorite at room temperature and circum-neutral pH conditions can be estimated to be on the order of 10^–17^ mol cm^−2^ s^−1^. Considering a typical value of *ρ* = 3.0 g cm^−3^ for chlorite and a molar mass of *M* = 697 g mol^−1^, ∆*h* is on the order of 0.1 Å, i.e., much less than an atomic monolayer at the chlorite surface. These crude calculations illustrate that Mn mobilization through the dissolution of chlorite with a circum-neutral pH fluid is highly unlikely. Therefore, an alternative mechanism to explain this mass transfer requires the existence of a microenvironment with greater weathering properties, such as that provided by microbial biofilm.

Several studies have demonstrated that microenvironments can be generated at the silicate-microbe contact^23^, where the local conditions in terms of pH and saturation state strongly differ from the bulk conditions^24,25^, with the development of surface biofilms further intensifying this effect through hydraulic decoupling^26^. Although the large-scale impact of chemical compounds secreted by microbes on silicate weathering rates remains an open and controversial question (e.g.^27–30^), several studies showed that chemically aggressive conditions (low pH, high concentration of organic acids) can result in a significant increase of silicate weathering rates, at least locally^25,31^. Here, an increase of the dissolution rate of chlorite by up to two orders of magnitude would have been required to get an appreciable release of Mn. According to the dissolution rate law developed by Lowson et al.^20^, such an increase can be reached if the local pH conditions in the vicinity of chlorite are on the order of 3, a value that is fully compatible with pH measured in some microbial biofilms in previous studies^24^.

The microorganisms are the major catalysts of manganese cycling in the natural environment^32^ and manganese is a micronutrient essential for the development of microbial communities, for which rocks represent the main source^33^. As such, it might have been targeted by microbes for several reasons, which include Mn oxidation by chemolithoautotrophs^32–34^ or incorporation as enzyme cofactor^35^.

One can wonder whether (i) the borers specifically targeted areas where microbes were already thriving at the surface of the siltstone and actively dissolving the crystals, or (ii) whether attachment of macroborers was a prerequisite to the establishment of microbial communities dissolving the siltstone. Supporting the first assertion, a few studies have proposed that microborings supposedly attributed to microbial weathering (e.g.,^36^) might weaken rocky substrates, eventually facilitating the subsequent drilling of microborings by bivalves^14^. However, all occurrence of silicate microborings that we are aware of dealt with volcanic rocks and more specifically, pre-fissured basalt glass^15,36,37^. As a matter of fact, our multiscale investigation of the rock substrate did not reveal the presence of any tubular microchannels, and biofilms were not observed anywhere other than in macroborings. As a consequence, we speculate that a nascent bioabrasion of the substrate by the bivalves was required to allow for the establishment of microbial communities and trigger the onset of microbial weathering. Supporting this assertion, freshwater mussels are known to concentrate limiting nutrients such as C, N and P in the benthos and stimulate biofilm growth (^38^ and references therein). In turn, microbially-induced rock weathering likely contributed to a greater dissolution along grain boundaries, ultimately facilitating grain detachment and rock-boring by *Lignopholas fluminalis*. Of note, this mechanism would be the biotic equivalent of the abiotic erosion and weathering of limestone^39^.

To conclude, our study sheds new light on the possible mechanisms of silicate bioerosion by macroborers. On the one hand, we suggest that microorganisms likely benefited from the early stages of siltstone drilling by macroborers to thrive at the bottom of macroborings. On the other hand, we provide evidence that microbes contributed to bioerosion by actively dissolving minerals, while hard minerals (quartz and feldspars) trapped in biofilms at the surface of the shells further facilitated the development of macroborings via mechanical abrasion. Therefore, the association between *Lignopholas fluminalis* and microbes has the main characteristics of what is commonly defined as a symbiotic action. Finally, this finding also raises three main concluding remarks:

(i) In addition to the increase in macrofaunal diversity previously reported^7^, the development of macroborings also likely contributed to an unexpected increase of microbial diversity that remains largely unexplored;
(ii) Our study underlines that preventive strategies to mitigate bioerosion might have to target on suppression of bacterial biofilm development in order to achieve effective solutions;
(iii) Finally, although the contribution of microbes to silicate weathering at large space and time scales remains unknown and debated, the present study suggests that this impact is far from negligible when coupled to macroborers in what appears as a symbiotic relation. As suggested here, such microbial communities may contain specific microorganisms with efficient weathering-ability, which would be worth investigating to possibly identify efficient bioinspired strategies of silicate weathering, of prime importance for a range of industrial and societal concerns including CO_2_ sequestration.

## Materials and methods

### Sample description

A detailed description of the sampling site, rock substrate and rock-boring species can be found in Bolotov et al.^8^. In brief, the samples were collected in a freshwater environment in the middle reaches of Kaladan River: 21.0094° N, 92.9813° E, altitude of 11 m above sea level, Rakhine State, western Myanmar. The samples were deposited in the Russian Museum of Biodiversity Hotspots [RMBH], Federal Center for Integrated Arctic Research of the Ural Branch of the Russian Academy of Sciences, Arkhangelsk, Russia.

The rock substrate was classified as a siltstone (primary grain size of 2–62 μm). A mean microindentation hardness (Vickers test) value of the substrate rock is 0.62 GPa with a range of 0.50–0.72 GPa. The main rock-forming minerals consist of quartz (30 wt%), clay minerals (32–47 wt%), feldspars (8–15 wt%), and chlorite (7–9 wt%). The macroborings from the Kaladan River were identified by Bolotov et al.^8^ as to correspond to the ichnospecies *Gastrochaenolites anauchen* Wilson & Palmer, 1998.

The freshwater boring species was determined by Bolotov et al.^8^ as *Lignopholas fluminalis* on the basis of morphological characters. Phylogenetic analyses conducted in this previous study suggested that *Lignopholas fluminalis* is related to the marine piddock species *Barnea davidi*, and the ancestral area reconstruction models suggest that the most recent common ancestor of the *Lignopholas* + *Barnea* clade was a marine bivalve. Finally, an assemblage of nestling species was also found in the macroborings, consisting of macroinvertebrates including clams, gastropods, polychaetes and a sponge^8^.

In addition, non-borer bivalves (*Scaphula deltae*; Arcidae) originated from the same Kaladan River were also investigated in the present study to provide a baseline to distinguish between the chemical compositions of the shells of non-borers species compared to the shells of *Lignopholas fluminalis*.

### Electron microscopy characterizations of the siltstone substrate

The rock sample was first cut with a diamond blade saw (~ 10 cm on a side) across two macroborings of interest (Fig. 1) to facilitate further handling for microscopy observations, which were conducted either with a TESCAN VEGA II SEM equipped with an EDAX PEGASUS energy dispersive X-ray (EDX) spectrometer operated at LHyGeS (Strasbourg, France) or with a Field Effect Gun (FEG) SEM ZEISS ULTRA55 equipped with an EDX system from Bruker operated at IMPMC (Paris, France). Three different locations were selected for detailed nanoscale characterizations (Fig. 1): (i) an area devoid of macroborings; (ii) the bottom of a large macroboring, immediately after removal of the inhabiting bivalve, and finally, (iii) the bottom of a smaller macroboring which did not preserve a bivalve. The first location was selected to document the mineralogical and chemical composition of an area representative of the substrate before boring. Conversely, the bottom of the two macroborings was selected to possibly identify discrepancies between the surface on an abandoned burrow and a macroboring occupied by a bivalve until the collection of the sample.

The rock and shell samples were then carbon-coated, and ultrathin electron transparent cross sections were subsequently prepared by FIB milling using the FEI HELIOS 600 NANOLAB dual-beam operated at CP2M (Marseille, France) following methods previously described by Daval et al.^40^. In brief, FIB Ga^+^ ion milling was carried out at an ion beam voltage of 30 kV and beam currents ranging from 9 nA to 90 pA for the final steps. Micrometer-thick sections were lifted out in situ using an Omniprobe 200 micromanipulator and transferred to a half copper grid for final ion milling to electron transparency (final thickness of ~ 100 nm). This milling was performed at a reduced acceleration voltage of 5 kV to reduce beam damage. For the same reasons, the final cleaning steps were then operated at 2 and 1 kV.

TEM and scanning transmission electron microscopy (STEM) observations were performed on FIB foils using a 200 kV JEOL 2100F microscope operated at IMPMC (Paris, France) equipped with a field emission gun. EDX spectra were acquired in STEM mode to probe the chemical composition of the imaged materials, with a focused electron beam (1 nm) and a detection limit close to 0.1 wt%. The analyses were conducted on a total of four FIB thin sections (two in the “pristine” area and one in each selected macroboring).

### Microscopic observations and chemical analyses of the shells

Independently, detailed analyses of the contact zone between the rock and a bivalve from another macroboring were performed using a combination of SEM–EDX with electron backscatter diffraction (EBSD), following methods previously described by Gabitov et al.^41^. In brief, the analyses were conducted with a SEM (JSM-6480LV, JEOL) equipped with an EDX spectrometer (X-Max^n^, Oxford Instrument) and an EBSD system (NordlysMax^2^) operated at Moscow State University, Russia. Polished cut samples were coated with 35 nm of carbon. Analyses were conducted at 20 kV accelerating voltage, 0.7 nA probe current and count rate about 17 kcps (with dead time about 22–25%) during 100 s live time. Program INCA (version 21b, “Oxford Instruments”) with XPP-correction model was used for processing of EDX spectra. Identification of the space group of carbonates was conducted using EBSD. For the analysis of the diffraction patterns and processing the results, software HKL (Oxford Instruments) and Inorganic Crystal Structure Database (ICSD) were used.

The elementary compositions of four shells of borers *Lignopholas fluminalis* and four shells of non-borers *Scaphula deltae* from the same Kaladan River were compared after acid digestion of organic-free carbonate component of the shells following Bolotov et al.^42^. For this, the entire shells were rinsed in MilliQ water and ground in an agate mortar. Acid digestion of the ground shells was performed by treating them in H_2_O_2_, HNO_3_, HNO_3_ + HCl and, finally, HNO_3_ at 80 °C in Teflon containers placed in individual evaporation boxes (class A 100) located inside a clean room (class ISO A 10,000). This allowed dissolving only the carbonate and organic part of the shells without attacking the possible silicate admixtures. The digestion products were evaporated to dryness, redissolved in 10% HNO_3_ and diluted by a factor of 5,000 for major and trace elements analysis using an AGILENT 7500CE ICP MS. Three-point calibration against a standard solution of known concentration (1, 10 and 100 ppb) was realized, using indium and rhenium as internal standards to correct for instrumental drift and possible matrix effects.

The efficiency of the acid digestion protocol and analysis was checked using the international geostandard for carbonate sediments (CJT-1). The measurement uncertainties basically ranged from 5 to 10% at 1–1,000 µg/L to 20–30% at 0.001–0.1 µg/L. In the latter case, elevated uncertainties resulted from the high dilution factor (from 1,000 to 5,000) of the starting samples. For samples with very low concentrations of trace elements (~ 0.001 µg/L, close to the detection limits), the minimal estimated uncertainty was 30%.

## Supplementary information

Supplementary Information 1.

## Data Availability

Data are available from the corresponding author upon request.
