# Supplementary material for: Symbiotic cooperation between freshwater rock-boring bivalves and microorganisms promotes silicate bioerosion
Source: Sci Rep. 2020 Aug 7;10:13385. doi: 10.1038/s41598-020-70265-x (PMC7415154; doi:10.1038/s41598-020-70265-x)
Supplement: Supplementary file 1 — Supplementary Information 1. [file 41598_2020_70265_MOESM1_ESM.pdf]

Supplementary materials for:

Symbiotic cooperation between freshwater rock-boring bivalves and microorganisms promotes silicate bioerosion

Damien Daval[1,*], François Guyot[2,3], Ivan N. Bolotov[4], Ilya V. Vikhrev[4], Alexander V. Kondakov[4], Artem A. Lyubas[4], Andrey Y. Bychkov[5], Vasily O. Yapaskurt[5], Martiane Cabié[6] and Oleg S. Pokrovsky[7,8]

Corresponding author: Damien Daval, email: ddaval@unistra.fr

This PDF file includes:

Figures S1 to S4

Tables S1 to S3

SI References

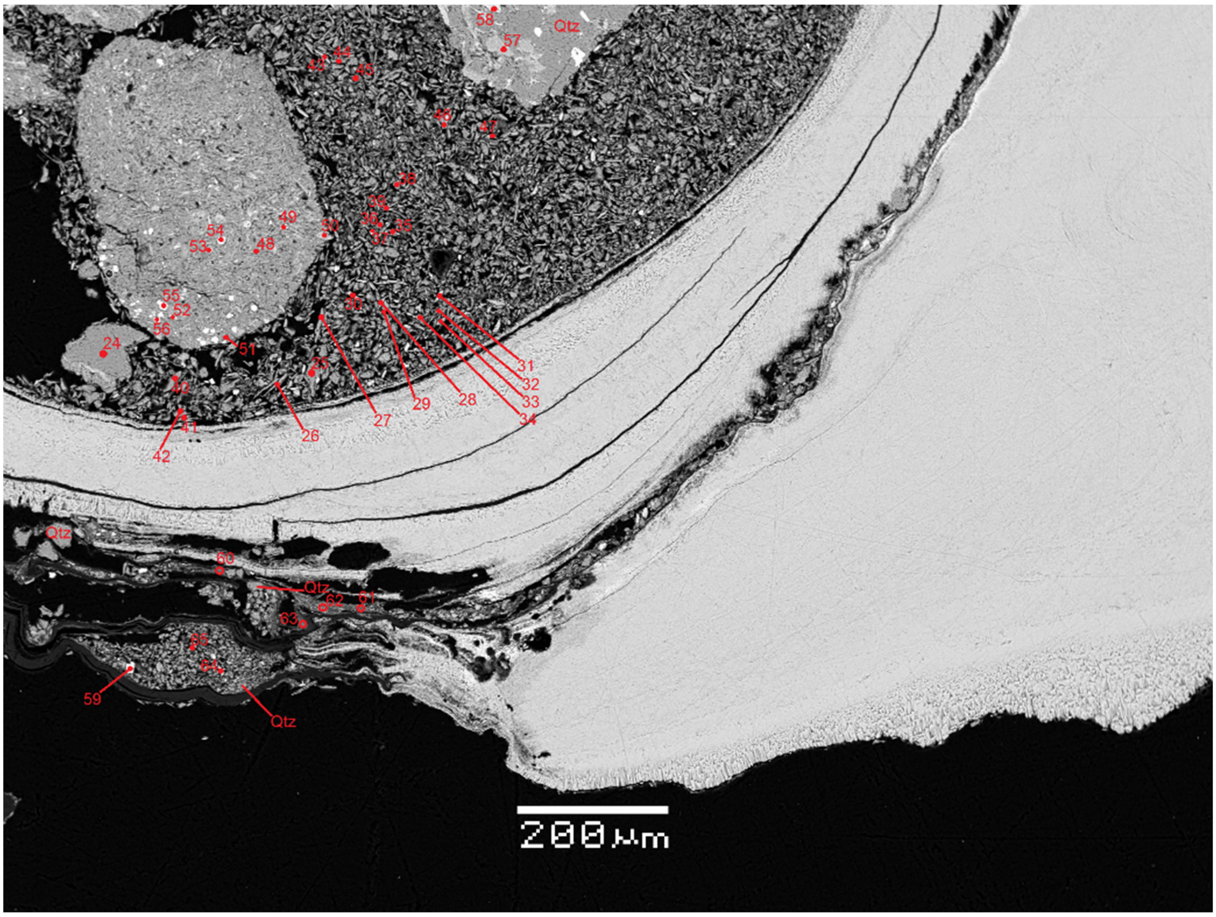

Fig. S1. (a) BSE image of the shell cross-section showing where aragonite has been analyzed (red spots). The “abrasive” minerals embedded into the organic film at the interface between the shell and the siltstone are essentially quartz, feldspars and sericite. See Table S1 for details.

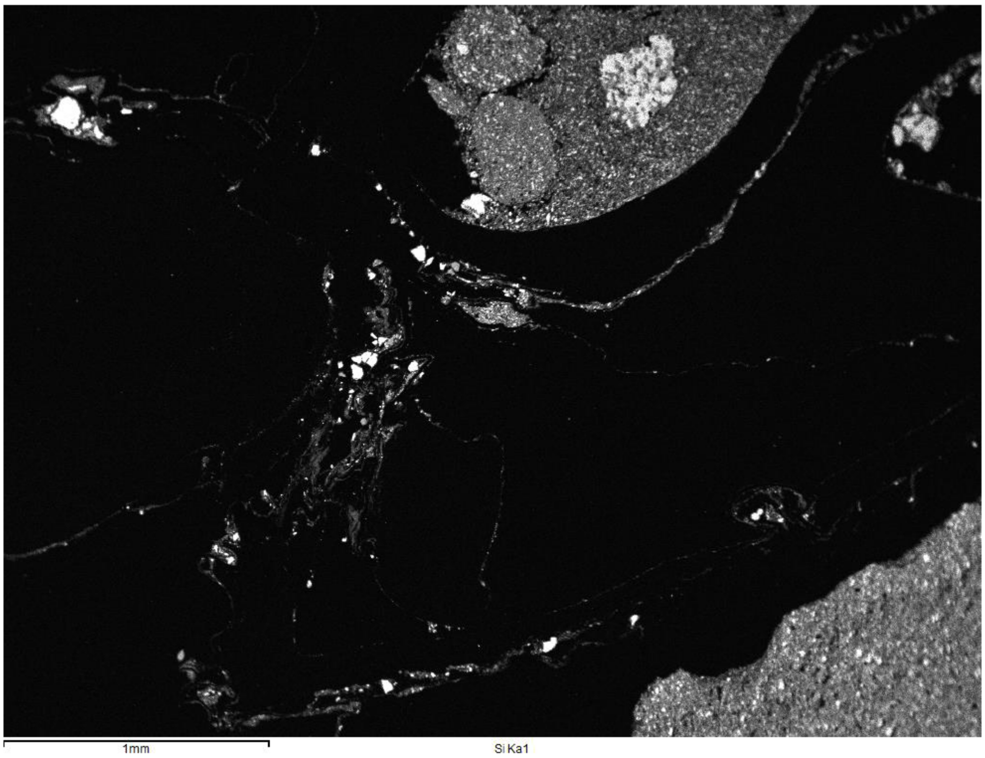

Fig. S2. Close-up view of the organic films showing Si distribution. The organic films contain abundant debris of quartz grains.

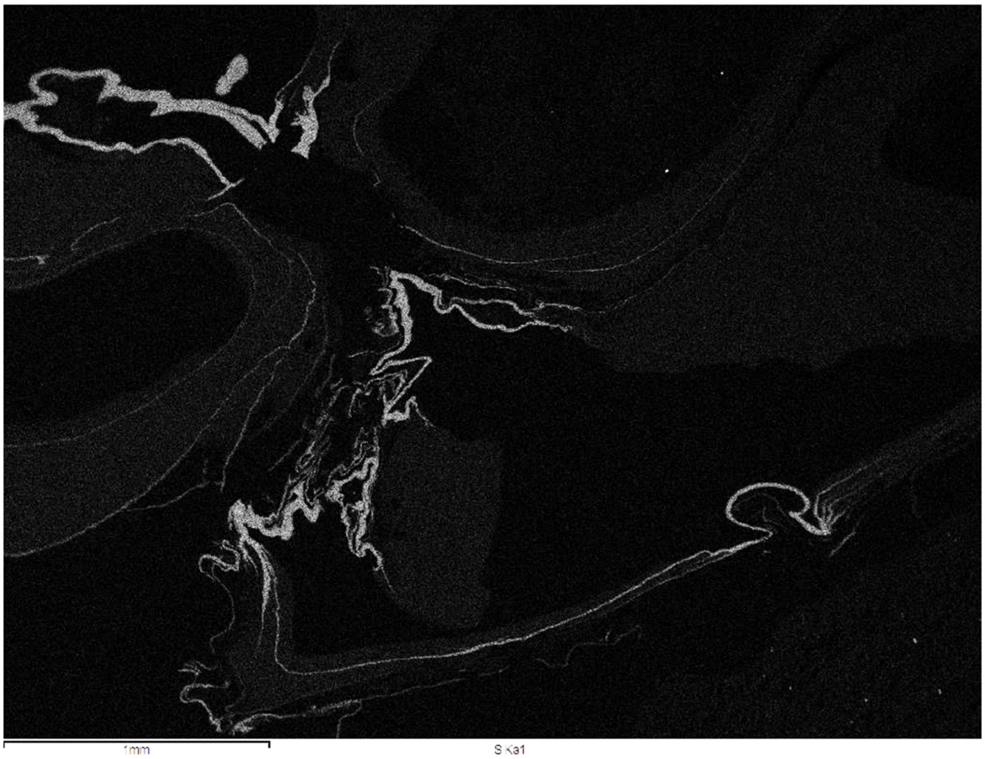

Fig. S3. Cross-section of the shell of a bivalve collected in one of the macroborings. The organic films are easily detectable in elementary maps of N and S, as can be seen in this image which represents the S distribution. High S concentration in the organic film might result from the presence of cysteine.

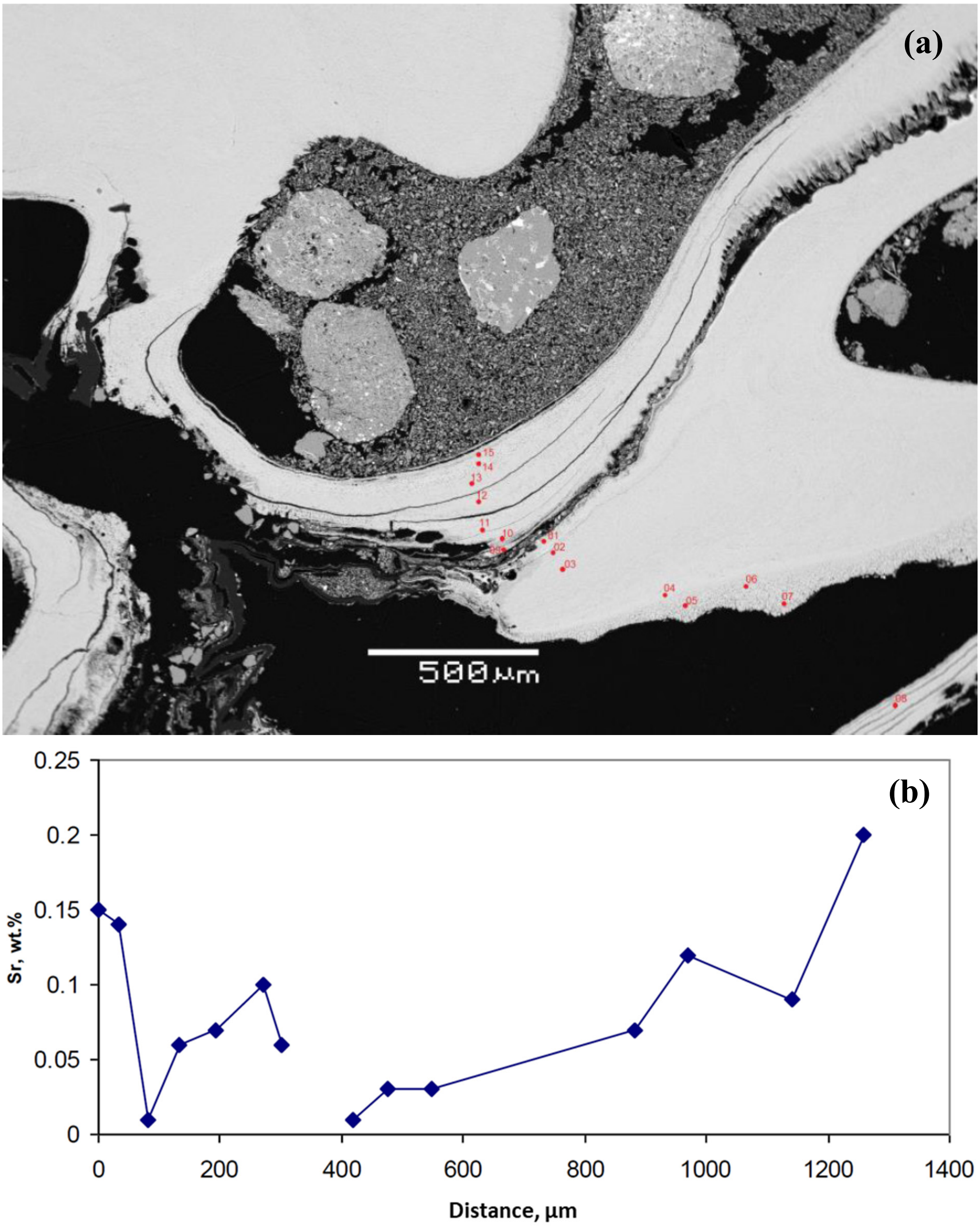

Fig. S4. (a) BSE image of the shell cross-section showing where aragonite has been analyzed (red spots). The chemical composition of aragonite is very homogenous, although slightly enriched in Sr at the extreme point of the shells. (b) Sr composition profile (points 15 to 07 in Fig. S4a) across the shell outer surface. The detection limit of Sr is 0.01 %; the other impurities were not detected. See details of method in Gabitov et al.[1].

Table S1. Chemical composition of minerals present within the organic matrix and the siltstone determined by SEM-EDS with EBSD at Moscow State University. Location of datapoints is shown in Fig. S1.

Sampling point	Mineral	weight % of elements (Oxygen by stoichiometry)													
		Na	Mg	Al	Si	P	S	K	Ca	Ti	Mn	Fe	Ba	O	Total
24	Qtz				46.2									52.63	98.83
25	Qtz				46.69									53.2	99.89
26	Ser	0.69	0.53	17.62	20.76	0		7.08	0.19	0.19	0	1.18		41.89	90.12
27	Ser	0.64	0.82	18.97	21.63	0		7.69	0.07	0.25	0	1.08		44.36	95.51
28	Chl+Ser	0.67	3.61	15.13	18.91	0.04		2.96	0.19	0.39	0.08	10.28		41.58	93.87
29	Ser	0.03	0.57	18.22	23.18	0.05		7.1	0.28	0	0	1.6		45.09	96.11
30	Chl	0.05	6.92	13.23	11.57	0.03		0.2	0.16	0.06	0.26	19.61		35.4	87.48
31	Chl	0.06	5.68	11.5	12	0.02		0.16	0.2	0	0.68	22.87		34.55	87.72
32	Ser	0.53	0.21	18.87	20.85	0.02		7.18	0.12	0.14	0	0.86		42.73	91.5
33	Ser	0.14	0.77	15.58	20.11	0		7.77	0.09	0.41	0	2.19		39.84	86.89
34	Ser	0.35	0.53	15.86	17.67	0.02		6.02	0.11	0.13	0.06	2.08		36.71	79.55
35	Ser	0.16	0.79	15.47	19.44	0		7.48	0.1	0.25	0	2.13		38.84	84.67
36	Ser	0.32	0.7	18.28	21.06	0.03		5.08	0.25	0	0	1.48		42.44	89.65
37	Chl	0.09	4.77	12.85	12.69	0.03		0.33	0.13	0.11	0.18	23.03		35.93	90.13
38	Chl+Ser	0.13	1.31	9.48	21.38	0.02		2.85	0.23	1.19	0	4.07		36.37	77.04
39	Ser	0.14	1.15	12.67	18.28	0.03		6.69	0.15	0.2	0	3.68	0.64	35.62	79.24
40	Ser	0.85	0.66	17.22	21.37	0.04		6.42	0.13	0.23	0	0.89		42.22	90.03
41	Chl	0.06	3.45	9.3	13.57	0.1	0.03	0.24	0.71	0.18	0.27	13.66		30.71	72.42
42	mica	0.35	0.39	12.19	30.03	0		4.68	0.17	0.12	0	0.78		46.76	95.46
43	Chl	0.03	8.54	12.61	12.47	0.03		0.6	0.09		0.14	14.97		35.58	85.06
44	Chl+Ser	0.12	0.76	12.52	18.31	0.08		2.95	0.25	0.54	0.07	5.46		35.29	76.35
45	Chl+Ser	0.19	0.51	11.03	14.84	0.15		1.83	0.26	0.25	0.07	5.28		29.49	63.9
46	Ser	0.24	0.82	16.57	20.46	0.05		7.42	0.15	0.35	0	2.02		41.12	89.19
47	Chl	0.05	4.5	8.52	13.01	0.08		0.51	0.51	0.31	0.34	16.83		30.91	75.57
48	Chl+Ser	0.25	0.79	14.49	17.78	0.24		4.09	0.21	0.48	0.09	5.6		36.94	80.96
49	Chl	0	6.37	11.6	12.13	0.08		0.28	0.17	0.02	0.37	21.57		34.86	87.44
50	Kfs	0.42	0	10.45	30.11	0.03		13.22	0.11	0		0.23	0.3	46.63	101.49
51	Chl+Ser	0.18	1.53	14.42	20.74	0.19		4.61	0.27	0.74	0.08	6.39		41.16	90.3
52	Chl	0.08	4.71	12.15	11.88	0.07		0.27	0.15	0.06	0.37	22.97		34.4	87.11
53	Chl	0.08	4.31	7.3	15.14	0.18		0.65	0.4	0.07	0.41	21.74		33.52	83.79
54	limonite	0	0.11	0.14	1.75	0.45	0.09	0.09	0.38		0.37	53.92		18.64	75.94
55	limonite	0.06	0.2	1.28	1.5	0.93	0.14	0.07	0.46		0.45	49.94		19.04	74.06
56	limonite	0	0.17	0.42	1.92	0.56	0.09	0.12	0.44	0.03	0.38	53.11		19.07	76.3
57	Kfs	0.53		10.64	30.31	0.05		13.2				0.2	0.17	47.03	102.14
58	Chl	0	5.88	12.43	11.36	0.05		0.32	0.14	0.04	0.22	22.63		34.63	87.69
59	TiO2 (Rt)		0.05	0.45	0.63			0.17	0.1	56.75		0.48		39.41	98.37
60	micas-mix	0.22	0.82	7.77	18.2	0.08	0.11	2.01	0.44	0.22	0.08	3.3		30.22	63.45
61	micas-mix	0.4	0.74	6.48	19.6	0.12	0.03	1.62	0.83	0.23	0.03	2.26		30.4	62.73
62	micas-mix	0.33	1.28	9.45	16.2	0.12	0.04	2.5	1.57	0.23	0.04	4.56		30.63	66.94
63	micas-mix	0.59	0.64	6.08	17.48	0.06	0.09	1.5	1.07	0.24	0.05	2.26		27.72	57.8
64	Chl	0.13	5.51	12.79	11.96	0.09	0.02	0.12	0.19	0.08	0.26	23.53		35.8	90.49
65	Ser	0.93	0.28	20.24	21.87	0.08	0.02	7.23	0.21	0.15	0	0.8		45.45	97.25

Abbreviations: Qtz, quartz; Chl, chlorite; Ser, sericite; Kfs, K-feldspar.

Table S2. Chemical composition of organic films attached to the shell (C is calculated by mass balance).

N	O	Al	Si	S	Cl	K	Ca	Fe	Sr	C
21.3	22.32	0.11	0.02	4.13	0.05	0.03	0.15	0.02	0.04	51.79
22.81	18.66	0.11	0.03	4.15	0.02	0.02	0.14	0.06	0.02	53.93
20.84	21.08	0.1	0.04	4.01	0.03	0.02	0.16	0.04	0.03	53.61

Table S3. Ratio of element concentration in the shells of borers (M137, *Lignopholas fluminalis*) to non-borers (M152, *Scaphula deltae*). The values were averaged on analyses performed on four individual shells of each species, with typical uncertainty ranging from 10 to 30% due to sizable variability among individuals. Significant ($p < 0.05$) differences in the ratio are indicated by asterisk.

ratio M137:M152

0.70	Li	0.75	Zr
0.79	Be	0.99	Mo
3.90*	B	0.30	Cd
0.85	Na	0.78	Sb
0.76	Mg	0.75	Cs
0.63	Al	1.56*	Ba
0.79	Si	0.97	La
0.53	P	0.90	Ce
0.99	S	0.91	Pr
0.63	K	0.82	Nd
0.95	Ca	0.94	Sm
0.95	Ti	0.86	Gd
0.73	V	0.92	Tb
0.66	Cr	0.84	Dy
0.66	Mn	0.75	Ho
0.73	Fe	0.79	Er
0.77	Co	0.80	Tm
0.66	Ni	0.94	Yb
0.99	Cu	0.37*	Hf
0.72	Zn	0.64	Ta
0.60	Ga	0.24*	W
0.54	Ge	0.68	Tl
0.59	As	0.92	Pb
0.69	Rb	0.79	Th
0.99	Sr	0.37*	U
0.77	Y		

SI References

1. Gabitov, R., Sadekov, A., Yapaskurt, V., Borrelli, C., Bychkov, A., Sabourin, K. & Perez-Huerta, A. Elemental Uptake by Calcite Slowly Grown From Seawater Solution: An in-situ Study via Depth Profiling. *Front. Earth Sci.* **7**. doi: 10.3389/feart.2019.00051(2019)
